# NTCP Calculations of Five Different Irradiation Techniques for the Treatment of Thymoma

**DOI:** 10.3390/curroncol30080561

**Published:** 2023-08-19

**Authors:** Michalis Mazonakis, Stefanos Kachris, Maria Tolia, John Damilakis

**Affiliations:** 1 Department of Medical Physics, Faculty of Medicine, University of Crete, 71003 Iraklion, Greece; john.damilakis@med.uoc.gr; 2 Department of Radiotherapy and Oncology, University Hospital of Iraklion, 71110 Iraklion, Greece; leukisgr@yahoo.com (S.K.); mariatolia@uoc.gr (M.T.)

**Keywords:** thymoma, 3D-CRT, IMRT, VMAT, NTCP

## Abstract

This study provided normal tissue complication probability (NTCP) calculations from photon radiotherapy techniques in eleven patients with thymoma. Five plans were created for each participant using three-dimensional conformal radiotherapy (3D-CRT), five-field intensity modulated radiotherapy (5F-IMRT), seven-field IMRT (7F-IMRT), and volumetric modulated arc therapy with full arcs (FA-VMAT) and partial arcs (PA-VMAT). The target coverage, homogeneity index and conformation number for the planning target volume (PTV) and dosimetric parameters for the organs-at-risk (OARs) were taken from the fifty-five generated plans. The patient-specific NTCP of the lungs, heart and esophagus was calculated with an in-house software tool using differential dose-volume histograms and the equivalent uniform dose model. The PTV dose metrics from 3D-CRT were inferior to those from IMRT and VMAT plans. The dose constraints for the OARs were met in all treatment plans. The NTCP range of the lungs, heart and esophagus was 0.34–0.49%, 0.03–0.06% and 0.08–0.10%, respectively. The NTCPs of the heart for the incidence of peridarditis from IMRT and VMAT were significantly smaller than those from conformal treatment (*p* < 0.05). The 7F-IMRT was significantly superior to FA-VMAT in reducing the NTCP of the lungs and the risk of pneumonitis (*p* = 0.001). Similar superiority of 5F-IMRT over PA-VMAT for lung protection was found (*p* = 0.009). The presented results may be employed in the selection of the appropriate irradiation technique for restricting the complications in the adjacent OARs.

## 1. Introduction

The epithelial tumors of the thymus gland include thymomas and thymic carcinomas. Most of these malignancies are thymomas mainly presented in the mediastinal region [1]. Thymomas are rare tumors with an incidence of approximately 0.15 cases per 100,000 person-years [2]. However, the thymomas are the most common malignant diseases in the anterior mediastinum [1]. Radiotherapy plays a major role in the effective management of these tumors. The administration of radiation therapy is recommended for patients with unresectable disease or incompletely resected thymoma [3]. Postoperative therapeutic irradiation may be also applied for subjects having a locally advanced disease [3].

Thymomas are usually diagnosed in subjects aged 50 to 60 years old [4]. The five-year survival rate of this malignancy has currently reached 90% [3]. For these middle-aged cancer patients with the good prognosis, it is of great importance to ensure the minimization of complications related to radiation therapy. This type of treatment always involves high energy beams delivering high doses to the tumor site. Parts of the adjacent radiosensitive organs, such as lungs, esophagus and heart, often receive high doses increasing the probability of toxicity including pneumonitis, pericardial effusions and esophageal stricture [5]. Patients with thymoma may be also subjected to an elevated risk for second cancer induction after treatment [6].

The determination of toxicity through the direct follow-up of irradiated patients is a difficult long-term process because of the rarity of thymomas. Several studies have provided theoretical estimates of the complications associated with the external-beam radiotherapy [7,8,9,10,11,12,13]. The vast majority of these reports were focused on the risk of radiation carcinogenesis [7,8,9,10,12]. The literature lacks information on the non-cancer adverse effects of radiation. Moiseenko et al. estimated the normal tissue complication probability (NTCP) only for lungs irradiated using a ^60^Co unit or X-rays with energy of 18–25 MV [11]. Computed tomography (CT) planning was performed only in 57% of the study participants. Yan et al. calculated the NTCP of the lungs and heart in patients with thymoma [13]. Their calculations were restricted to a single irradiation approach. It is well known that the radiation exposure of each organ-at-risk (OAR) may vary by the irradiation technique [5]. The variation of the organ-specific probability for toxicity by the applied treatment method needs to be investigated.

The aim of this study was to calculate the NTCPs of critical organs from three-dimensional conformal radiotherapy (3D-CRT), five-field intensity modulated radiation therapy (5F-IMRT), seven-field IMRT (7F-IMRT), full-arc volumetric modulated arc therapy (FA-VMAT) and partial-arc (PA) VMAT in patients with thymoma.

## 2. Materials and Methods

### 2.1. Patients

This retrospective planning study involved eleven patients (seven females and four males) with stage II or III thymoma who were previously irradiated in our Radiation Oncology department. The median patient age was 54.3 ± 12.8 years. Adjuvant radiation therapy with a tumor dose of 50.4 Gy was administered to five subjects after complete resection of limited stage II tumors. Postoperative treatment to 54 Gy was given to five patients with microscopically positive resection margins after excision of large stage II tumors or limited stage III tumors. One patient with gross residual disease invading the pericardium and the chest wall received a total dose of 59.4 Gy. The fraction dose per day was 1.8 Gy for all study participants. None of the patients received induction or adjuvant systemic therapy.

### 2.2. Treatment Planning

All participants in this study were subjected to a planning CT prior to irradiation. The CT scanning was performed with the patients in supine position. The contiguous 5 mm CT slices were transferred to a Monaco workstation for contouring and treatment planning (Monaco v. 5.11.03, Elekta AB, Stockholm, Sweden). The high accuracy of the dose calculation algorithms and the robustness of the optimization methods of the Monaco system have been previously discussed in the literature [14,15]. The planning target volume (PTV) included the whole thymus, surgical clips and the surrounding areas with residual disease. The lungs, spinal cord, esophagus and heart were manually contoured on the CT scans. The contouring was carried out by a senior radiation oncologist experienced in treating patients with thoracic malignancies.

Five different treatment plans were generated for each patient using photon beams emitted by a medical linear accelerator (Infinity, Elekta AB, Stockholm, Sweden). The above therapy machine is equipped with Agility MLC head having 160 leaves of projected width 5 mm at the isocenter. The first three-dimensional conformal radiotherapy (3D-CRT) plan consisted of four coplanar radiation fields with gantry angles of 0°, 90°, 180° and 270°. This plan was designed using 10 MV photon beams. The dose calculations were carried out using the collapsed cone algorithm. Ninety-five percent of the prescribed dose had to cover at least 95% of the PTV in the 3D-CRT plans. A low photon beam energy of 6 MV was used for IMRT and VMAT planning. Static 5F and 7F arrangements were employed for IMRT plans. The gantry angles for the 5F-IMRT were 275°, 317°, 0°, 42° and 85° whereas those for 7F-IMRT were 0°, 51°, 102°, 153°, 204°, 255° and 306°. The maximum number of control points per beam was set to 20. The VMAT technique was used for the two additional plans of each patient. Double full-arc (FA) VMAT plans were initially generated. This treatment was designed to be applied at two coplanar 360° arcs in clockwise and counterclockwise rotations. Dual PA-VMAT plans rotating in opposite directions were also created. The length of the partial arc was 170° starting at a fixed gantry angle of 275°. The dual-arc approach is currently applied for VMAT planning in our department [16,17,18]. The dosimetric calculations for all IMRT and VMAT plans were performed with a Monte Carlo algorithm. The aim of these plans was to give the prescribed dose to more than 95% of the target volume. The dose constraints for the lungs, esophagus, heart and spinal cord used for 3D-CRT, IMRT and VMAT planning are presented in Table 1 [1]. Similar dose constraints have already been applied for postoperative radiotherapy for thymoma [19]. All treatment plans in this study were generated by a medical physicist with clinical experience in this field of more than 15 years.

### 2.3. Planning Dose Parameters

The target coverage (TC), homogeneity index (HI) and conformation number (CN) were calculated for all fifty-five treatment plans. The TC, which refers to the proportion of the volume of the PTV receiving the prescribed dose ${\;(\mathrm{PTV}}_{\mathrm{PD}})$ to the whole target volume, was found with the formula:

(1) $$\mathrm{TC}=\frac{{\mathrm{PTV}}_{\mathrm{PD}}}{\mathrm{PTV}}\;\times \;100\%$$

The homogeneity index (HI) was calculated as follows:

(2) $$\mathrm{HI}=\frac{D_{5\%}}{D_{95\%}}$$

where ${\;D}_{5\%}$ and $D_{95\%}$ are the radiation doses to 5% and 95% of the PTV, respectively. The conformation number (CN) was calculated with the aid of the following equation:

(3) $$\mathrm{CN}=\frac{{{(\mathrm{PTV}}_{\mathrm{PD}})}^{2}}{\mathrm{PTV}\;\times \;{\mathrm{TV}}_{\mathrm{PD}}}$$

where ${\mathrm{TV}}_{\mathrm{PD}}$ is the total volume of the patient’s body absorbing the prescribed dose.

The maximum dose (D_max_) to the spinal cord and the average dose (D_av_) of the two lungs, esophagus and heart were recorded from each treatment plan. Moreover, the V_5Gy_ and V_20Gy_ of the lungs, the V_50Gy_ of the esophagus and the V_30Gy_ of the heart were obtained by cumulative dose volume histograms (DVHs). The parameter V_iGy_ denotes the volume of the OAR receiving a radiation dose exceeding iGy.

### 2.4. Radiobiological Parameters

The equivalent uniform dose (EUD)-based model was employed to determine the NTCP for the heavily irradiated OARs [20,21]. An in-house software tool developed using Python 3.8 was employed for these calculations [22]. Data from differential DVHs directly exported by the treatment planning system could be easily retrieved by the software. The software automatically calculated the NTCP value in less than 40 s. The NTCP for pneumonitis, pericarditis and perforation/clinical stricture due to the unavoidable exposure of the lungs, heart and esophagus, respectively, were calculated with the aid of the software. The EUD for each of the above OARs was found using the formula:

(4) $$\mathrm{EUD}={\left[\sum_{i=1}\left(V_{i}{\;\mathrm{EQD}}_{i}^{\alpha }\right)\right]}^{a^{-1}}$$

where V_i_ is the organ volume receiving a biological equivalent physical dose of EQD_i_ and α is a dimensionless organ-specific parameter. The EQD_i_ was calculated as follows:

(5) EQDi=Diαβ+Di,fractionαβ+2

where  αβ is a linear quadratic parameter for each different exposed organ tissue and ${\;D}_{\mathrm{fraction}}$ is the tumor dose per fraction. The NTCP calculations were carried out using the following equation:

(6) $$\mathrm{NTCP}={\left[1\;+\;{\left(\frac{{\mathrm{TD}}_{50}}{\mathrm{EUD}}\right)}^{4\gamma_{50}}\right]}^{-1}$$

where TD_50_ is the tolerance radiation dose associated with a complication probability of 50% and γ_50_ is a unitless organ-specific model parameter related to the slope of the dose–response curve. The parameters employed for calculating the NTCP of the lungs, heart and esophagus are shown in Table 2 [22].

### 2.5. Statistical Analysis

Box and whisker plots were created to present the distribution of the NTCPs for lungs, heart and esophagus calculated from the five radiotherapy techniques. To appraise the NTCP differences between the treatment planning strategies, a Wilcoxon matched pairs test was applied. A *p*-value below 0.05 was considered to indicate a statistically significant difference. The above statistical analysis was performed with the software Graph Pad Prism version 4.0 (Graph Pad Software, San Diego, CA, USA).

## 3. Results

### 3.1. Planning Dose Parameters

**Figure 1:**
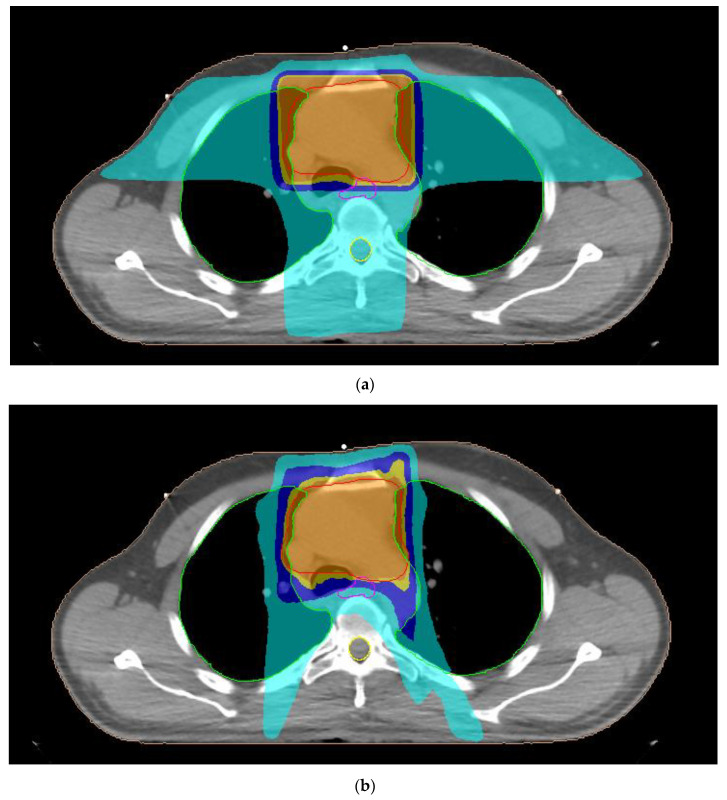

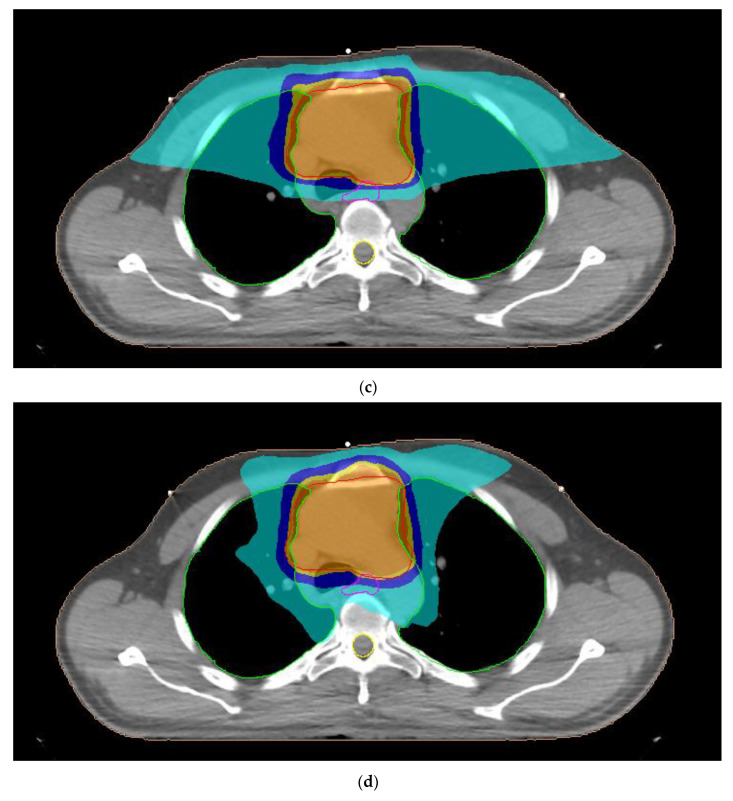

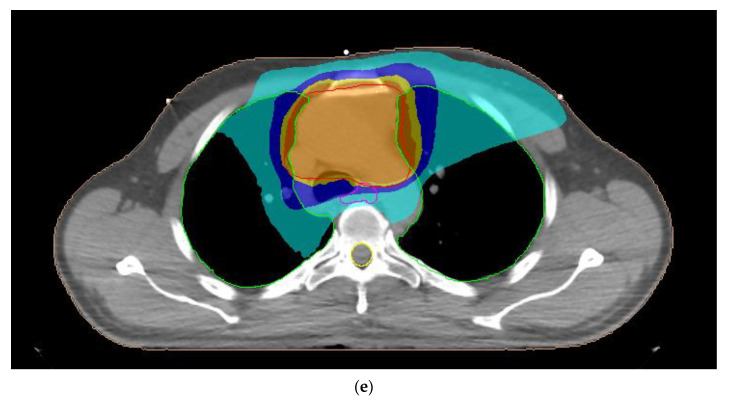
Typical dose distribution around the target volume, shown in red color, for (**a**) three-dimensional conformal radiotherapy, (**b**) seven-field intensity modulated radiation therapy, (**c**) five-field intensity modulated radiation therapy, (**d**) full-arc volumetric modulated arc therapy and (**e**) partial-arc volumetric modulated arc therapy techniques of a male patient with thymoma. The 95%, 90%, 75% and 45% isodoses are presented with orange, dark yellow, dark blue and light blue colors, respectively. The lungs, esophagus and spinal cord are denoted by the green, pink and yellow contours, respectively.

A typical dose distribution of a patient with thymoma from 3D-CRT, 7F-IMRT, 5F-IMRT, FA-VMAT and PA-VMAT plans is presented in Figure 1. The dose parameters for the PTV are summarized in Table 3. The mean TC, HI and CN for 3D-CRT were 58.4%, 1.11 and 0.48, respectively. The corresponding parameters for IMRT plans reached 95.6%, 1.06 and 0.83 whereas those for VMAT plans were up to 95.7%, 1.07 and 0.82.

The planning parameters for the surrounding OARs are shown in Table 4. The mean value of the D_av_ of the lungs varied from 10.9 to 11.4 Gy for the five different techniques. The V_20Gy_ for 3D-CRT was 26.3% and reduced to 20.9–21.9% for the IMRT and VMAT plans. In contrast, the V_5Gy_ for plans with intensity modulated techniques was 46.7–49.3% whereas that from 3D conventional treatment was decreased to 45.8%. The range of the D_av_ of the heart and esophagus from both IMRT and VMAT plans was 8.5–8.7 Gy and 16.5–17.0 Gy, respectively. The above organ doses with the 3D-CRT were higher and equal to 10.2 Gy and 18.9 Gy. The V_30Gy_ of the heart was 14.8% for conformal treatment and it was reduced to 10.9–11.5% with the use of IMRT and VMAT. The V_50Gy_ values for esophagus ranged from 6.1% to 6.8% for the five examined treatment techniques. The mean value of the D_max_ to the spinal cord due to IMRT and VMAT was 17.9 Gy. The corresponding dose due to 3D-CRT increased to 30.6Gy. 

### 3.2. Radiobiological Parameters

**Figure 2:**
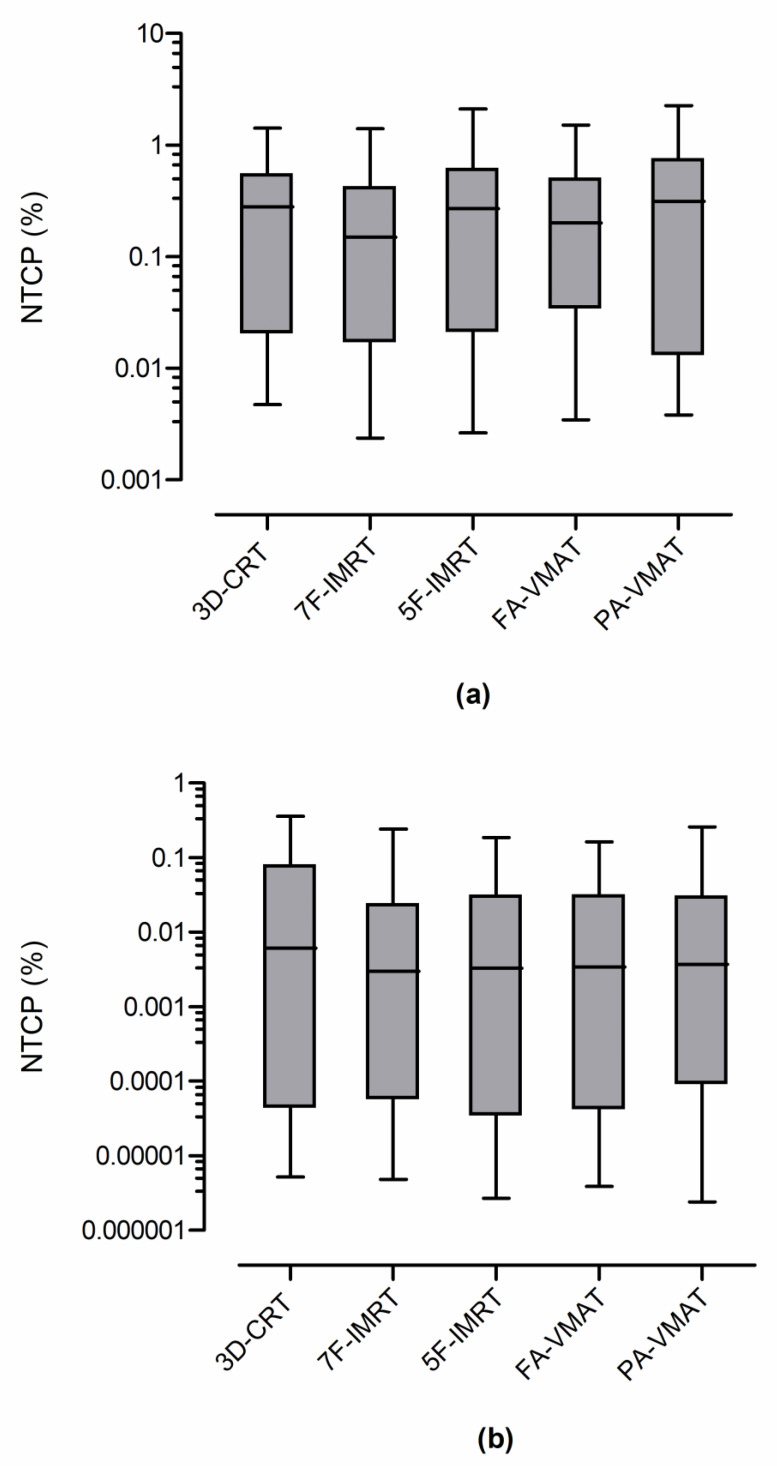

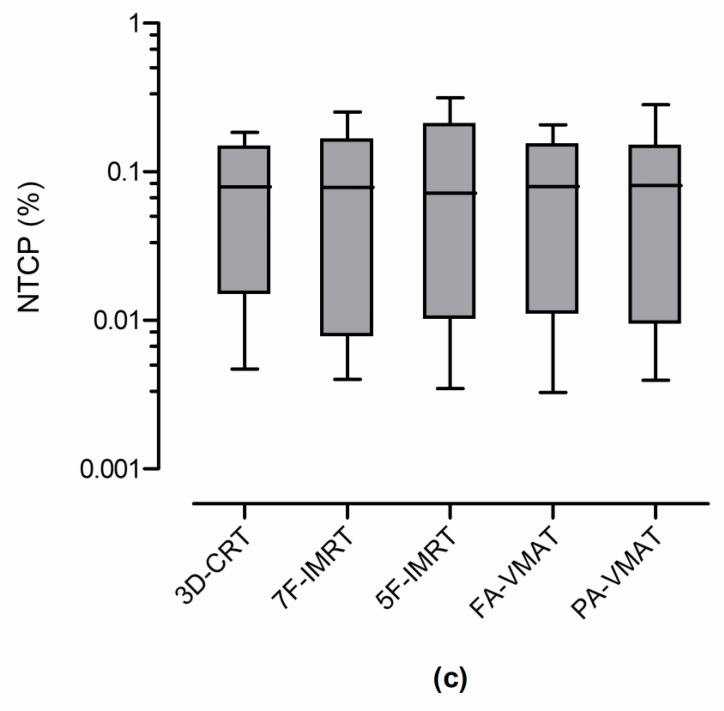
Box and whisker plots showing the normal tissue complication probability (NTCP) calculations on a logarithimic scale for (**a**) lungs, (**b**) heart and (**c**) esophagus derived from three-dimensional conformal radiotherapy (3D-CRT), seven-field intensity modulated radiation therapy (7F-IMRT), five-field intensity modulated radiation therapy (5F-IMRT), full-arc volumetric modulated arc therapy (FA-VMAT) and partial-arc volumetric modulated arc (PA-VMAT). Each box ranges from the 25th percentile to 75th percentile. The line inside the box indicates the median value. The whiskers extend to the minimum and maximum values.

The average NTCP (NTCP_av_) for the examined OARs as derived from the five different radiation therapy techniques are shown in Table 5. The 3D-CRT resulted in an NTCP_av_ for the lungs, heart and esophagus of 0.38%, 0.06% and 0.08%, respectively. The corresponding NTCPs due to IMRT and VMAT were 0.34–0.49%, 0.03–0.04% and 0.09–0.10%. The NTCP calculations for the three examined OARs are shown in the box and whiskers plots of Figure 2. The widest variation in the NTCP values of the lungs was observed in the PA-VMAT plans. The corresponding variation for the heart and esophagus was presented in 3D-CRT and 5F-IMRT plans, respectively. The statistical differences between the NTCPs derived from the five irradiation approaches are summarized in Table 6. Significant differences were observed between the NTCPs of the lungs obtained by the 7F-IMRT and PA-VMAT plans (*p* = 0.001). This was also observed between 5F-IMRT and PA-VMAT (*p* = 0.009). The NTCPs of the heart derived from 3D-CRT plans were statistically different from those calculated by IMRT and VMAT plans (*p* = 0.005–0.042). No significant difference was observed for all other comparisons.

## 4. Discussion

Five different irradiation techniques for thymoma, including the conventional 3D-CRT and the advanced 5F-IMRT, 7F-IMRT, PA-VMAT and FA-VMAT, were compared in this study. A four-field arrangement was used for generating the plans with the 3D-CRT technique. Similar field configurations comprising three to five beams have been employed during conformal treatment of thymoma [10,19]. Two-dimensional and 3D radiation techniques based on a simple arrangement with two anteroposterior and posteroanterior opposed beams have also been applied for treating thymic tumors [5,10].

All generated treatment plans satisfied the dose constraints for the lungs, heart, esophagus and spinal cord. They also provided the required dose coverage to the target volume. Both IMRT and VMAT systematically led to a better dose homogeneity and conformity within the target in respect to 3D-CRT. A superior target coverage was also observed with the modern intensity modulated techniques. All fifty-five generated treatment plans satisfied the dose constraints for the OARs and they were considered acceptable for clinical use. The IMRT and VMAT plans reduced the D_av_ of the esophagus, the D_max_ of the spinal cord, the V_20Gy_ of the lungs and the dose parameters of the heart compared to the values derived from the 3D-CRT. The 3D-CRT resulted in lower V_5Gy_ of the lungs than that from the intensity-modulated techniques. Similar average lung doses and V_50Gy_ values for esophagus were observed between the five irradiation techniques.

Small differences were found in the dose parameters of the PTV and OARs as derived from the treatment plans generated with the four advanced intensity-modulated techniques. For example, the TC and HI calculated by the PA-VMAT plans were slightly better than those from FA-VMAT, although the two techniques led to the same CN value. A small increase in V_20Gy_ and V_5Gy_ of the lungs from FA-VMAT in respect to the parameters associated with PA-VMAT was observed. The opposite result was found for the average cardiac dose and the parameter V_30Gy_ of the heart. The above differences could be attributed to several factors related to the application of the specific dose constraints for generating the IMRT and VMAT plans, the selection of the beam configuration for each technique and the performance of the optimization algorithms employed for the process of treatment planning.

Yan et al. used the 7F-IMRT free-breathing techniques with 3 mm internal target volume margin [13]. They reported a D_av_ of the lungs and esophagus of 10.28 and 17.54 Gy, respectively. These values are similar to those found in our group of patients. However, they gave a D_av_ of the heart of 15.90 Gy which is higher than that presented here. Our dose results were much lower than those given by Haefner et al. [19]. They reported that the D_av_ of the lungs, heart and esophagus from 3D-CRT plans was equal to 26.91, 24.76 and 36.19 Gy, respectively. The corresponding doses from VMAT were 21.57, 17.08 and 28.75 Gy. The results of the current study were elevated compared to those derived from the IMRT and VMAT plans in the work of He et al. [23]. They gave a D_av_ of the lungs, heart and esophagus of up to 9.33, 5.56 and 15.12 Gy. A wide variation in the D_max_ of the spinal cord from 6.33 Gy to 60.63 Gy due to the use of 3D-CRT, IMRT and VMAT in patients with thymoma has been reported in the literature [13,19,23]. The corresponding spinal dose in our study reached 17.9 Gy with static and rotational intensity modulated radiotherapy and was 30.6 Gy with conventional conformal treatment. It should be noted that the dosimetric data of the above previous studies corresponded to a stable target dose of 50 Gy [13,19,23]. Our results were taken with PTV doses from 50.4 to 59.4 Gy depending onthe patient’s disease stage.

The evaluation of radiation therapy plans should not be based only on dosimetric analysis but also on the determination of radiobiological indices [24,25]. In the present study, the NTCPs of the lungs, heart and esophagus due to thymoma irradiation were calculated in terms of pneumonitis, pericarditis and perforation or stricture, respectively. These calculations were made on the basis of the EUD determined with the aid of the Niemierko model [20,21]. The NTCPs of the esophagus and heart calculated from DVHs obtained by the five treatment techniques were found to be rather small. The average NTCP of the heart was 0.06% for 3D-CRT and reduced to 0.03–0.04% for IMRT and VMAT. The corresponding average values for esophagus were 0.08–0.10%. It should be mentioned that the maximum NTCP value for the heart was 0.36% for a patient with thymoma subjected to 3D-CRT, whereas that for esophagus reached 0.31% during IMRT with a five-field arrangement. The average NTCP of the lungs varied from 0.34% to 0.49% by the treatment method applied for the management of thymoma. These values were higher than those for heart and esophagus. The NTCP of the lungs exceeded 1% only in two patients due to the large dimensions of the target resulting in a heavy radiation exposure of these critical structures. The relatively increased NTCPs of the lungs should not be ignored by radiation oncologists and medical physicists in the process of treatment planning for thymomas.

The patient-specific NTCPs were statistically compared for the three examined OARs between the five different radiation therapy techniques. Some of these comparisons led to significant differences for the radiosensitive lungs and heart (*p* < 0.05). The NTCPs of the heart from 3D-CRT were significantly increased than those derived from 5F-IMRT, 7F-IMRT, FA-VMAT and PA-VMAT. Moreover, the 7F-IMRT significantly reduced the NTCP of the lungs compared to FA-VMAT. A similar considerable decrease was found when 5F-IMRT was used instead of the PA-VMAT. Insignificant NTCP differences between the treatment techniques were found for the esophagus.

This study may be limited by the relatively low number of participants irradiated for thymoma. This was due to the rarity of this malignant disease [2]. The current work compared radiobiological parameters calculated from five photon radiotherapy techniques which may be applied in patients with thymoma. Hardron therapy may also be employed for the management of thymic epithelial tumors [26]. Further research is needed to compare the NTCP values presented here with those from proton and carbon ion therapy.

## 5. Conclusions

Radiotherapy plans were created for patients with thymoma using 3D-CRT, 5F-IMRT, 7F-IMRT, FA-VMAT and PA-VMAT techniques. All generated plans were found to be clinically acceptable. Treatment planning data were employed to calculate the NTCP of the lungs, heart and esophagus. The 5F-IMRT, 7F-IMRT, FA-VMAT and PA-VMAT were significantly superior to the 3D-CRT in decreasing the NTCP of the heart and the subsequent risk for pericarditis. No significant differences were observed in the NTCP of the lungs and esophagus as calculated by the conventional conformal treatment and the advanced intensity modulated techniques. The current study provides useful information about the probability for developing complications in heavily irradiated organs due to the administration of radiation therapy for thymoma with five different techniques.

## Figures and Tables

**Table 1:** Dose constraints used for the generation of treatment plans for thymoma.

Organ-at-Risk	Parameter
Lungs	D_av_ < 20 Gy
	V_20Gy_ < 35%
	V_5Gy_ < 65%
Heart	D_av_ < 26 Gy
	V_30Gy_ < 45%
Esophagus	D_av_ < 34 Gy
	V_50Gy_ < 50%
Spinal cord	D_max_ < 45 Gy

D_av_: average dose; V_iGy_: organ volume receiving more than iGy; D_max_: maximum dose.

**Table 2:** Parameters used for the calculation of normal tissue complication probability.

Organ-at-Risk	α	γ_50_	TD_50_ (Gy)	α/β (Gy)	Endpoint
Lungs	1	2	24.5	3	Pneumonitis
Heart	3	3	50	2.5	Pericarditis
Esophagus	19	4	68	3	Perforation/Stricture

**Table 3:** Dose metrics for the planning target volume, presented as mean values ± one standard deviation, from five radiation therapy techniques for thymoma.

Technique	TC (%)	HI	CN
3D-CRT	58.4 ± 10.7	1.11 ± 0.02	0.48 ± 0.10
7F-IMRT	95.6 ± 0.6	1.06 ± 0.01	0.83 ± 0.03
5F-IMRT	95.3 ± 0.4	1.06 ± 0.01	0.82 ± 0.03
FA-VMAT	95.4 ± 0.6	1.07 ± 0.01	0.82 ± 0.03
PA-VMAT	95.7 ± 0.4	1.06 ± 0.01	0.82 ± 0.02

3D-CRT: three-dimensional conformal radiotherapy; 7F: seven fields; 5F: five fields; IMRT: intensity modulated radiation therapy; FA: full arc; PA: partial arc; VMAT: volumetric modulated arc therapy; TC: target coverage; HI: homogeneity index; CN: conformation number.

**Table 4:** Dose parameters for the organs-at-risk, presented as mean values ± one standard deviation, from five radiation therapy techniques for thymoma.

Organ-At-Risk	Parameter	3D-CRT	7F-IMRT	5F-IMRT	FA-VMAT	PA-VMAT
Lungs	D_av_ (Gy)	11.4 ± 2.7	10.9 ± 2.9	11.0 ± 2.9	11.3 ± 2.8	11.3 ± 3.0
	V_20Gy_ (%)	26.3 ± 6.4	20.9 ± 7.4	21.5 ± 7.5	21.9 ± 7.1	21.2 ± 6.2
	V_5Gy_ (%)	45.8 ± 11.3	46.7 ± 11.2	47.8 ± 12.5	49.3 ± 12.3	48.8 ± 13.2
Heart	D_av_ (Gy)	10.2 ± 6.0	8.6 ± 5.3	8.5 ± 5.2	8.6 ± 5.1	8.7 ± 5.7
	V_30Gy_ (%)	14.8 ± 11.1	11.4 ± 8.4	10.9 ± 8.2	10.9 ± 7.9	11.5 ± 8.8
Esophagus	D_av_ (Gy)	18.9 ± 5.1	16.8 ± 4.2	16.6 ± 4.2	17.0 ± 4.2	16.5 ± 4.3
	V_50Gy_ (%)	6.1 ± 6.9	6.4 ± 6.0	6.8 ± 5.9	6.4 ± 5.7	6.1 ± 5.3
Spinal cord	D_max_ (Gy)	30.6 ± 3.7	17.9 ± 1.0	16.6 ± 1.2	17.5 ± 0.6	17.5 ± 0.9

3D-CRT: three-dimensional conformal radiotherapy; 7F: seven fields; 5F: five fields; IMRT: intensity modulated radiation therapy; FA: full arc; PA: partial arc; VMAT: volumetric modulated arc therapy; D_av_: average dose; V_iGy_: organ volume receiving more than iGy; D_max_: maximum dose.

**Table 5:** Normal tissue complication probability (NTCP), presented as mean value ± one standard deviation, from five radiation therapy techniques for thymoma.

Technique	NTCP (%)		
	Lungs	Heart	Esophagus
3D-CRT	0.38 ± 0.44	0.06 ± 0.11	0.08 ± 0.07
7F-IMRT	0.34 ± 0.46	0.04 ± 0.07	0.09 ± 0.09
5F-IMRT	0.39 ± 0.62	0.03 ± 0.06	0.10 ± 0.11
FA-VMAT	0.43 ± 0.57	0.03 ± 0.06	0.09 ± 0.07
PA-VMAT	0.49 ± 0.72	0.04 ± 0.07	0.09 ± 0.09

3D-CRT: three-dimensional conformal radiotherapy; 7F: seven fields; 5F: five fields; IMRT: intensity modulated radiation therapy; FA: full arc; PA: partial arc; VMAT: volumetric modulated arc therapy.

**Table 6:** Statistical comparison of the normal tissue complication probability (NTCP) calculations derived from five radiation therapy techniques for thymoma.

Techniques	*p*-Value		
	Lungs	Heart	Esophagus
3D-CRT vs. 7F-IMRT	0.083	0.007 *	0.320
3D-CRT vs. 5F-IMRT	0.240	0.019 *	0.320
3D-CRT vs. FA-VMAT	0.517	0.042 *	0.413
3D-CRT vs. PA-VMAT	0.700	0.005 *	0.320
7F-IMRT vs. 5F-IMRT	0.765	0.966	0.083
7F-IMRT vs. FA-VMAT	0.001 *	0.966	0.517
7F-IMRT vs. PA-VMAT	0.206	0.206	0.577
5F-IMRT vs. FA-VMAT	0.465	0.700	0.413
5F-IMRT vs. PA-VMAT	0.009 *	0.831	0.123
FA-VMAT vs. PA-VMAT	0.966	0.577	0.517

3D-CRT: three-dimensional conformal radiotherapy; 7F: seven fields; 5F: five fields; IMRT: intensity modulated radiation therapy; FA: full arc; PA: partial arc; VMAT: volumetric modulated arc therapy. * Statistically significant differences (*p* < 0.05).

## Data Availability

Data of this study are available by the corresponding author upon reasonable request.
